# Mobile applications for atrial fibrillation self-management: A systematic search and evaluation

**DOI:** 10.1016/j.hroo.2026.06.015

**Published:** 2026-07-02

**Authors:** Wojciech Losos, Carrie Shao, Biqi Wang, Spyros Kitsiou, Ben S. Gerber, David D. McManus

**Affiliations:** 1 Department of Medicine, University of Massachusetts Chan Medical School, Worcester, Massachusetts; 2 Program in Digital Medicine, UMass Memorial Medical Center, Worcester, Massachusetts; 3 Department of Medicine, University of Illinois College of Medicine, Chicago, Illinois

**Keywords:** Atrial fibrillation, Mobile applications, Self-management, Mobile health, mHealth, App evaluation, Mobile Application Rating Scale, Shared decision-making

## Abstract

**Background:**

Atrial fibrillation (AF) is the most common cardiac arrhythmia, increasing the risk of stroke, heart failure, and healthcare costs. Although patient self-management can improve outcomes, sustaining long-term engagement is difficult. Mobile health applications may help address this gap, but their quality and clinical alignment have not been systematically assessed using a validated framework.

**Objective:**

This study aimed to systematically identify and evaluate AF self-management applications using the Mobile Application Rating Scale (MARS) and to assess how well these apps align with evidence-based practices for AF.

**Methods:**

We conducted a structured search of the Apple App Store and Google Play Store (June 2024, updated January 2025) for English-language, patient-focused apps supporting AF self-management (eg, symptom logging, medication reminders, and educational content). Eligible apps offered ≥1 self-management feature and either explicitly referenced AF or, for general self-management apps, provided features directly applicable to AF self-care. 2 reviewers independently rated app quality using the MARS across 4 domains (Engagement, Functionality, Aesthetics, and Information).

**Results:**

Of the 455 apps identified, 5 met all inclusion criteria. Common features included symptom tracking and medication logging, but coverage of guideline-recommended care domains varied. Mean MARS scores ranged from 4.07 to 4.53 (out of 5). Higher-performing apps excelled in functionality and information quality but often lacked features such as stroke-risk assessment and personalized feedback.

**Conclusion:**

Current AF self-management apps show high MARS quality but lack comprehensive, clinically grounded functionality, highlighting a gap in digital tools for AF self-care. Improved codesign processes and standardized evaluation frameworks may guide development of more effective apps.

**Key Findings**

▪ A systematic search of the Apple App Store and Google Play Store yielded only 5 free, English-language atrial fibrillation (AF) self-management applications from 455 apps identified, revealing a sparse landscape of dedicated tools.
▪ Overall app quality on the Mobile Application Rating Scale (MARS) was high (mean 4.07–4.53 of 5), driven mainly by functionality and information quality.
▪ Despite high MARS scores, no app offered comprehensive, guideline-concordant functionality; stroke-risk assessment, personalized feedback, and structured lifestyle support were frequently absent.
▪ Inter-rater agreement was only moderate (Krippendorff’s α = 0.53; highest app α = 0.69), so comparative quality rankings should be interpreted cautiously.
▪ Because most self-management apps fall outside active Food and Drug Administration device regulation and app-store reviews do not verify clinical accuracy, clinician guidance, curated digital formularies, and codesign are needed to integrate high-quality apps into AF care.

## Introduction

Atrial fibrillation (AF) is the most prevalent cardiac arrhythmia globally, significantly increasing the risk of stroke, heart failure, and healthcare costs.^1^ With an aging global population and a rising prevalence of chronic diseases, effective AF management has become a major public health priority.^2^ Patient self-management—including medication adherence, symptom monitoring, rhythm tracking, and lifestyle behavior adjustments (eg, heart-healthy diet, physical activity, and smoking cessation)—can play a critical role in controlling AF and improving clinical outcomes.^3^

Notably, landmark trials of goal-directed weight management and adjunctive lifestyle and pharmacologic weight loss have demonstrated substantial reductions in AF burden with sustained weight loss and comprehensive lifestyle modification.^4^^,^^5^ Although structured education and clinical support can improve adherence and quality of life in the short term, maintaining long-term patient engagement remains a persistent challenge.^3^^,^^6^

In response to these challenges, mobile health (mHealth) technologies—including smartphone applications integrated with wearable devices—offer the potential to support continuous rhythm monitoring, medication adherence, and lifestyle modification.^7^^,^^8^ However, the quality and clinical utility of AF-focused apps remain highly variable. A prior review by Turchioe et al^9^ examined mobile applications for AF detection and management, highlighting the diversity of available tools and the growing role of digital health in this space. Their review included both patient- and clinician-facing apps and focused largely on rhythm detection capabilities, without evaluating app quality using a validated framework.

Although some prior work has evaluated AF-related apps, including some with management features, no study to date has systematically assessed patient-facing AF self-management applications using a validated tool, such as the Mobile Application Rating Scale (MARS), or examined their alignment with evidence-based self-care practices. Our study addresses this gap by focusing specifically on tools designed to support patient engagement in AF self-management—such as symptom tracking, medication adherence, and lifestyle modification—and applying a structured rating scale to evaluate app quality across multiple domains.

Despite the growing number of digital tools, the evidence remains fragmented, and patients lack clear guidance on trustworthy, clinically grounded self-management options. Therefore, this study aimed to systematically identify, characterize, and evaluate patient-facing AF self-management apps using the validated MARS, and to assess how well these apps align with evidence-based care practices.

## Methods

The research reported in this paper adhered to the Preferred Reporting Items for Systematic Reviews and Meta-Analyses guidelines.

### Search strategy and app identification

We systematically searched the Apple iOS App Store and Google Play Store in June 2024 and updated the search in January 2025 using the terms “atrial fibrillation,” “arrhythmia,” and “cardiac rhythm.”

Apps were included if they met all of the following criteria: (1) free to download and usable without a mandatory paid subscription—apps offering optional in-app purchases were eligible provided their core self-management features were accessible at no cost (eg, AFibLife); (2) available in English; (3) designed for patient (rather than clinician-only) use; (4) explicitly referenced AF or, for general (non–AF-specific) self-management apps, offered self-management functionality directly applicable to AF self-care; and (5) offered at least 1 self-management feature such as symptom logging, medication reminders, or lifestyle education.

Therefore, general self-management apps that were not explicitly labeled for AF were eligible only when they were free, English-language, and patient-facing and provided ≥1 feature (eg, symptom tracking, medication-adherence support, vital-sign monitoring, education) that could plausibly support AF self-care in real-world use. Health Storylines met these criteria. This strategy aligns with evidence that patients frequently use multi-condition apps to manage AF alongside other chronic conditions.^10^ To supplement the app-store search and ensure comprehensive coverage, we reviewed prior systematic reviews of cardiovascular and AF-focused mHealth apps^9^^,^^10^ to identify additional apps relevant to AF self-care not captured by our keyword strategy.

When an app was available on both iOS and Android platforms, we retained the iOS version to ensure consistency in evaluation. Similar versions of the same app (eg, “Pro” or “Lite” editions) were treated as duplicates, and only the most complete or commonly downloaded version was retained for screening. We excluded general heart-rate or rhythm monitors without self-management features, blood-pressure tracking apps, clinician-only platforms, and apps requiring institutional logins.

This systematic review was not registered, and no formal protocol was prepared before conducting the review.

### App characterization

For each included app, we extracted descriptive data, including app developer, version, release/update dates, platform availability, cost, main functions, supported languages, privacy disclosures, and wearable-integration capabilities. Data privacy and security information was extracted from each app’s store description and the linked privacy policy when available. A full legal review of user agreements was not conducted.

Application-level usage metrics (eg, cumulative download counts and number of user reviews) were not consistently retrievable across platforms: the Apple App Store does not publicly report download counts, the Google Play Store reports only broad install ranges, and user-review counts were sparse and not directly comparable between stores. Therefore, these metrics were not extracted (see Limitations).

### App quality assessment

App quality was evaluated using the MARS,^11^ which assesses 4 domains: Engagement (entertainment, interest, customization, interactivity, and target group), Functionality (performance, ease of use, navigation, and gestural design), Aesthetics (layout, graphics, and visual appeal), and Information (accuracy of app description, goals, quality and quantity of information, visual information, credibility, and evidence base). Each subcategory was rated on a 5-point Likert scale (1 = inadequate, 5 = excellent). Domain scores were averaged to calculate a composite mean quality score for each app. The full MARS scoring framework is provided in Supplemental Appendix 1.

### Reviewer training and reliability

Two independent reviewers completed a calibration session using 3 non-study apps to standardize MARS scoring before independently rating each app. Discrepancies were resolved through consensus to determine final scores. Inter-rater reliability was quantified using Krippendorff’s alpha in R (version 4.3.3),^12^ treating item scores as ordinal variables. Krippendorff’s alpha is a robust reliability coefficient suitable for ordinal data and small sample sizes and has been widely adopted in health-technology evaluation studies.^13^ Per-app reliability results are reported in Supplemental Table 1.

Each application was rated on 18 MARS quality items spanning the 4 objective domains (Engagement: 5 items; Functionality: 4 items; Aesthetics: 3 items; and Information: 6 items). The Information-domain evidence base item was rated “not applicable” for all 5 applications, because none had been formally trialed in the published literature, and was therefore excluded from scoring and reliability calculations. Inter-rater reliability was accordingly computed across 90 pooled item ratings (18 items × 5 applications).

### Ethical considerations

This study was a systematic review and did not involve human participants. No identifiable personal data were collected or analyzed; therefore, institutional review board approval and informed consent were not required.

## Results

### Search results

**Figure 1:**
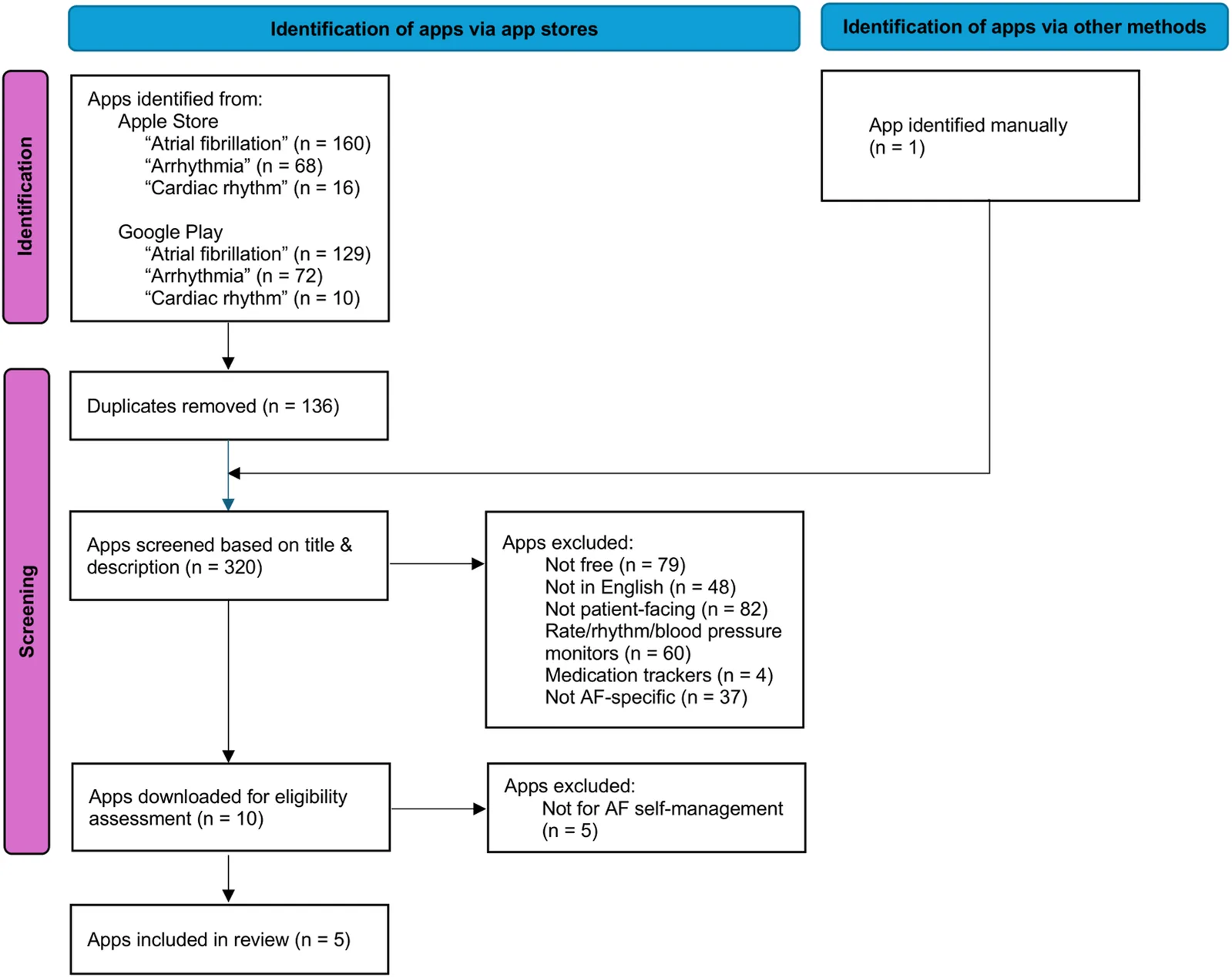
Preferred Reporting Items for Systematic Reviews and Meta-Analyses flow diagram of the app selection process. Systematic searches of the Apple App Store and Google Play Store identified 455 applications. After removal of duplicates and ineligible versions, 320 unique apps underwent title and description screening, of which 310 were excluded for not meeting the inclusion criteria. Ten apps were downloaded for full eligibility assessment, and 5 met all criteria for inclusion in the final analysis—4 identified through the app-store searches and 1 (Health Storylines) through the supplementary review of prior systematic reviews of cardiovascular and AF-focused mHealth apps.

A total of 455 apps were identified through systematic searches of the Apple App Store and Google Play. After removing duplicates and ineligible versions, 320 unique apps remained for title and description screening. Of these, 310 were excluded for not meeting inclusion criteria. 10 apps were downloaded for full eligibility assessment, and 5 met all criteria for final analysis. Four were identified through app-store searches, and 1 (Health Storylines) was identified through our supplementary review of prior systematic reviews. A summary of the selection process is provided in Figure 1.

### App characteristics and functionalities

Key app characteristics are summarized in Table 1. All 5 apps are available on iOS, and 4 are also available on Android. Each is free to download; 1 (AFibLife) offers optional in-app purchases that are not required to access core self-management features. Although not exclusively designed for AF, Health Storylines provides modular, customizable self-management tools—such as symptom tracking, medication logging, and a health diary—that can be adapted by patients with AF to monitor their condition and treatment adherence.

**Table 1:** Characteristics of included atrial fibrillation (AF) self-management apps

App Name	Developer	Platform	Version	Last Update	Cost	Primary Purpose	Data Privacy and Security	Connectivity and Integration	Features and Functionality
AFib Manager	@Point of Care	iOS	11.0.4	Oct 26, 2023	Free	AF management; data integration with Health app	Not described in public listing	Integrates with Health app; compatible with heart-rate devices	Heart-rate data integration; AF management tools (English)
AFib 2gether	Pfizer Inc.	iOS, Android	1.0.0	Jul 11, 2022	Free	Education; stroke-risk assessment; shared decision-making	Collects personal info from users and providers	Not specified	Stroke-risk questionnaire; educational resources; FAQ tool (English)
cliexa-PULSE	cliexa	iOS, Android	1.0.0	Jan 15, 2024	Free	Symptom tracking; medication management; episode logging	Not described in public listing	Not specified	Symptom tracking; medication logging; episode documentation (English)
AFibLife	Appmanschap B.V.	iOS	1.5.0	Jan 23, 2025	Free (in-app purchases)	AF education; QoL assessment; health-data integration	Data may be collected and linked to user identity	Option to connect health data from mobile device	AFEQT questionnaire; health-data integration; journaling; stroke-risk calculator (English, German, Japanese, Portuguese)
Health Storylines	Self Care Catalysts	iOS, Android	10.0.0	Oct 10, 2023	Free	Symptom tracking; medication adherence; health diary; customizable care plans	Data collected; user-controlled sharing	Syncs with Apple Health, Google Fit, some wearables	Symptom tracking; medication management; education; journaling; customizable trackers (English)

Developer, platform, version, update date, cost, primary purpose, and major features are summarized. AF = atrial fibrillation; AFEQT = Atrial Fibrillation Effect on Quality-of-Life questionnaire; QoL = quality of life.

Coverage of self-management domains varies across the apps (Table 2). AFib Manager offers comprehensive functionality, including symptom tracking, medication logging, episode recording, educational content, and health-device integration; however, it does not include stroke-risk assessment or shared-decision-making support. AFib 2gether focuses on education, stroke-risk communication, and decision support but lacks monitoring and tracking capabilities. cliexa-PULSE includes symptom tracking, medication management, and episode logging but does not provide educational content or stroke-risk tools. AFibLife supports all evaluated domains, although its medication feature is limited to logging only and does not include medication reminders, refill alerts, or integration with pharmacy or adherence-tracking tools. Health Storylines provides a customizable platform and medication tracking that, although not AF-specific, aligns with common self-care needs for patients with AF.

**Table 2:** Coverage of core AF self-management domains across included apps

App Name	Symptom Tracking	Medication Mgmt	Episode Logging	Education / Info	Stroke-Risk Assessment	Shared Decision-Making	Device Integration	Total Functions Covered
AFibLife	Yes	Partial (logging only)	Yes	Yes	Yes	Yes	Yes	6.5
AFib Manager	Yes	Yes	Yes	Yes	No	No	Yes	5
Health Storylines	Yes	Yes	No	Yes	No	No	Yes	4
cliexa-PULSE	Yes	Yes	Yes	No	No	No	No	3
AFib 2gether	No	No	No	Yes	Yes	Yes	No	3

Domains include symptom tracking, medication management, episode logging, education, stroke-risk assessment, shared decision-making, and device integration. Note: Partial functionality (eg, medication logging without reminders) was scored as 0.5.

### App quality assessment

Consensus MARS scores ranged from 4.07 to 4.53 (Table 3). AFib 2gether and AFibLife scored highest (4.53), followed by cliexa-PULSE (4.38). AFib 2gether had the highest Functionality score (5.00) and shared the highest Information-quality score (4.83). cliexa-PULSE had the highest Aesthetic score (5.00). Health Storylines received the highest Engagement score (4.40) but scored lower in Functionality (3.75) and Information-quality (4.17) scores, resulting in an overall score of 4.08. AFib Manager had the lowest mean score (4.07), primarily because of a lower Aesthetic rating (3.67). Apps that performed well across multiple MARS domains—particularly Functionality and Information—had the highest quality scores.

**Table 3:** Mean Mobile Application Rating Scale (MARS) scores across four quality domains

App	Engagement	Functionality	Aesthetics	Information	Mean App Quality
AFib Manager	4.20	4.25	3.67	4.17	4.07
AFib 2gether	3.60	5.00	4.67	4.83	4.53
cliexa-PULSE	4.20	3.50	5.00	4.83	4.38
AFibLife	4.20	4.75	4.33	4.83	4.53
Health Storylines	4.40	3.75	4.00	4.17	4.08

Domains include engagement, functionality, aesthetics, and information quality. Higher scores reflect better app performance; each subdomain is rated on a 1–5 scale.

### Inter-rater reliability

Overall inter-rater agreement was Krippendorff’s α of 0.53. Agreement varied across apps, ranging from α of 0.27 (AFib Manager) to α of 0.69 (Health Storylines), with intermediate values for the remaining apps (Supplemental Table 1).

## Discussion

### Principal findings

We found general acceptability but considerable variability in the quality and comprehensiveness of the 5 available AF self-management apps we reviewed. Although all included core features—such as symptom tracking and medication logging—none offered a fully integrated, guideline-aligned platform encompassing rhythm monitoring, patient education, behavioral support, and shared-decision-making tools. This highlights a critical gap in the availability of mHealth resources that comprehensively support AF self-management in line with evidence-based care. In particular, structured lifestyle interventions (eg, weight management, alcohol moderation), individualized stroke-risk education, and long-term adherence support were often absent or only partially addressed. Despite increasing interest in digital health, many AF apps still fall short of delivering clinically robust, user-centered functionality.^3^, ^4^, ^5^^,^^9^

The inclusion of a general-purpose platform, such as Health Storylines, underscores the practical reality that patients frequently adapt multi-condition self-management tools to support AF self-care alongside other comorbidities. Therefore, general apps may play a meaningful role in the AF digital-health landscape even when not explicitly AF-labeled.^10^

**Figure 2:**
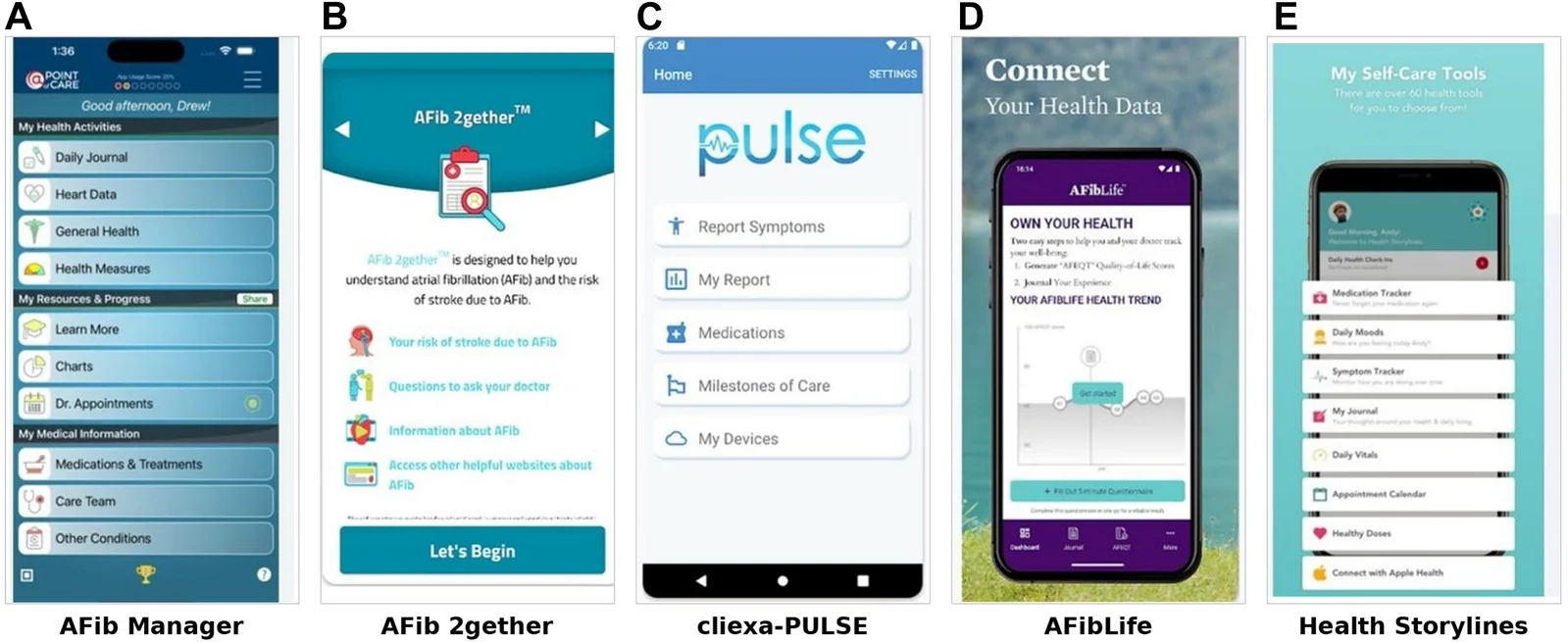
Representative user interfaces of the 5 included AF self-management applications. Representative screenshots of the primary self-management interface of each included app: **A:** AFib Manager: monitoring/data-integration dashboard; **B:** AFib 2gether: stroke-risk and shared-decision-making screen; **C:** cliexa-PULSE: symptom/episode logging menu; **D:** AFibLife: quality-of-life (AFEQT) assessment and health-trend view; **E:** Health Storylines: customizable self-care tracker. Screenshots reflect the app versions listed in Table 1; any displayed personal data are simulated.

Functionality and Aesthetic scores varied widely, reflecting persistent challenges in app usability and design, as illustrated by the differing interfaces of the included apps (Figure 2). These disparities likely stem from limited application of user-centered design principles, such as intuitive navigation, visual clarity, and mobile responsiveness—factors known to affect both adoption and sustained use.^11^ Superficial features alone are insufficient; meaningful engagement depends on seamless and accessible interfaces. Notably, even the highest-rated apps demonstrated limited personalization and dynamic feedback, mirroring broader trends in cardiovascular app development where tailored features remain underutilized despite their proven role in supporting behavior change, medication adherence, and self-efficacy.^10^^,^^14^

The variation in inter-rater agreement observed in this study (Supplemental Table 1) is itself informative: Health Storylines, which showed the highest agreement, has a modular design and clear labeling that may have facilitated more uniform interpretation, whereas apps with lower agreement often had less intuitive layouts or more ambiguous content. Integrating adaptive elements—such as customizable goals, personalized alerts, and feedback loops informed by user behavior—may help drive deeper engagement. Future research should explore how specific design features affect long-term adherence and whether embedding behavioral theory into app development can bridge the gap between initial interest and sustained use.

### Comparison with prior work

Our findings align with prior evaluations of cardiovascular mHealth tools, especially for heart-failure self-management, which have similarly reported wide variation in quality, limited use of behavioral frameworks, and inconsistent alignment with clinical guidelines.^10^ Many earlier reviews relied on narrative summaries or author-developed checklists, reducing reproducibility and comparability across studies. By contrast, our study used the validated MARS,^11^ providing a structured assessment across 4 domains—Engagement, Functionality, Aesthetics, and Information—and incorporated inter-rater reliability to enhance objectivity. This dual focus enabled us to differentiate apps not only by their content but also by the clarity and consistency of their user interface—a dimension often overlooked in prior reviews.^9^

### Clinical implications

For clinicians, these findings carry several practical implications. First, a high MARS score should not be read as evidence of clinical completeness: the highest-rated apps in our sample scored well on Engagement, Functionality, and Aesthetics yet omitted guideline-relevant functions such as stroke-risk assessment, personalized feedback, and structured lifestyle support. Therefore, clinicians should appraise an app’s specific feature set against the patient’s needs rather than relying on overall quality ratings or app-store prominence.

Second, the features most likely to be clinically meaningful map onto established AF care pathways, such as the Avoid stroke, Better symptom control, Cardiovascular risk and comorbidity management framework^2^: stroke-risk communication and decision support (best represented by AFib 2gether), symptom and episode logging that can inform rhythm- and rate-control decisions (cliexa-PULSE and AFib Manager), medication-adherence support, and education aligned with guideline-recommended lifestyle modification. Matching the app to the clinical task—for example, recommending a decision-support tool to facilitate anticoagulation discussions vs a tracking tool to characterize symptom–rhythm correlation—is more useful than seeking a single “best” app.

Third, because none of the evaluated apps integrated the full spectrum of guideline-concordant care, apps are best positioned as adjuncts that complement—rather than replace—clinical assessment, and are well suited for incorporation into shared decision-making for rhythm monitoring, anticoagulation, and lifestyle change. When recommending an app, clinicians should also consider its data-privacy disclosures and regulatory status (discussed below), particularly for tools that influence medical decisions.

These considerations are especially relevant for older adults with AF, who may face barriers such as low digital literacy, interface complexity, visual or motor impairment, and reduced confidence in using mobile tools.^15^^,^^16^ Selecting apps with clear, simple interfaces and offering brief in-clinic orientation may improve uptake in this population.

### Regulation and oversight of AF mobile applications

The regulatory environment helps explain why marketplace availability and high usability do not guarantee clinical accuracy. Under the US Food and Drug Administration’s Policy for Device Software Functions and Mobile Medical Applications, only software functions that meet the statutory definition of a medical device—and whose failure could pose a risk to patient safety—are actively regulated; the Food and Drug Administration exercises enforcement discretion or classifies as “general wellness” many lower-risk functions such as symptom logging, medication reminders, and patient education.^17^ Most of the self-management functions evaluated in this study fall outside active device regulation, whereas functions such as automated arrhythmia detection are more likely to require premarket review. Guidance updates issued in January 2026 further broadened the categories of low-risk wellness and clinical-decision-support software that fall outside active oversight,^17^ meaning many AF self-management apps will continue to reach patients without premarket evaluation of clinical accuracy.

App-store review processes provide a second, distinct layer of gatekeeping but are not designed to verify clinical validity. Apple subjects every submission and update to manual human review against its App Store guidelines, including health-specific requirements, whereas Google Play relies largely on automated, machine-learning–based review with selective manual escalation. These differences can affect app functionality, the frequency of updates, and the consistency of health-content scrutiny. Critically, neither process evaluates guideline concordance or clinical effectiveness, and the same companies act simultaneously as distributors, gatekeepers, and developers of competing health products—an arrangement that raises potential conflicts of interest.^18^

For clinicians and patients, the implication is that an app’s presence in a store, its polish, or its star rating cannot be assumed to reflect regulatory vetting or clinical accuracy. This reinforces the need for clinician guidance, curated digital formularies, and independent certification frameworks to help patients navigate a largely unregulated marketplace.

### Limitations

This study has several limitations. First, we restricted inclusion to free, English-language apps, which may have excluded high-quality paid or non-English options. For example, subscription-based apps, such as Kardia Premium, offer rhythm monitoring and patient education but were excluded owing to cost, or because they primarily focus on device integration rather than comprehensive self-management. The prevalence of such apps suggests our sample may not fully capture the broader mHealth landscape for AF. We also included Health Storylines, a general chronic-disease self-management app, to reflect how patients adapt multi-condition tools to support AF self-care in real-world practice.

Second, inter-rater reliability was only moderate overall (Krippendorff’s α = 0.53) and was based on a small number of MARS items per app; even the highest observed agreement (α = 0.69) remains in the low-to-moderate range. Therefore, comparative quality rankings among apps should be interpreted with caution, and small between-app differences in MARS scores may not be robust. Final scores were nonetheless determined by consensus, which mitigates the influence of individual rater variability on the reported values.

Third, although MARS provides a structured and validated framework,^11^ it may not fully capture real-world clinical relevance or patient usability; insights from patient feedback after extended use could reveal trust, engagement, and usability factors that structured appraisals alone may not detect.

Fourth, because mobile apps are frequently updated, features and design elements may have changed since our evaluation.

Fifth, we did not include direct clinician input in our app assessments, which could have added perspectives on content accuracy, workflow integration, and alignment with evidence-based care pathways.^10^

Sixth, application-level usage metrics (download counts and user-review counts) could not be reliably or comparably obtained across the Apple and Google platforms and were not analyzed. Consequently, we could not assess real-world adoption or popularity. Finally, our privacy and security assessment was limited to information provided in app-store descriptions and linked privacy policies; we did not conduct a detailed legal or technical audit of each app’s user agreement or data-handling practices.

## Conclusion

To ensure relevance, sustainability, and equity in AF self-management, future app development should prioritize interdisciplinary codesign involving clinicians, behavioral scientists, engineers, and patients. Codesign frameworks—such as participatory and human-centered design—can better align digital tools with user preferences and real-world needs, and engaging patients early through co-creation has been shown to improve usability, trust, and uptake.^14^^,^^15^ In summary, current AF self-management apps demonstrate high MARS quality but lack comprehensive, guideline-concordant functionality. Our findings underscore the need for greater transparency, standardization, and oversight in the development and evaluation of AF self-management apps. In the absence of active regulatory oversight, clinicians and patients must navigate a largely unregulated digital marketplace, increasing the risk of adopting tools with limited clinical accuracy or usability. Addressing this gap will require evidence-based frameworks and endorsement mechanisms—such as digital formularies or certification programs—and incorporation of app assessments into shared decision-making. Future research should examine how codesigned apps affect long-term patient engagement and clinical outcomes.

## Disclosures

The authors have no conflicts of interest to disclose.
