# Supplementary material for: Mobile applications for atrial fibrillation self-management: A systematic search and evaluation
Source: Heart Rhythm O2. 2026 Jul 2;7(9):1698–705. doi: 10.1016/j.hroo.2026.06.015 (PMC13594489; doi:10.1016/j.hroo.2026.06.015)
Supplement: Supplementary file 1 [file mmc1.docx]

**Supplemental Material**

*Losos W, Shao C, Wang B, Kitsiou S, Gerber BS, McManus DD. Mobile applications for atrial fibrillation self-management: A systematic search and evaluation. Heart Rhythm O².*

**Supplemental Table 1. Inter-rater reliability (Krippendorff’s α) by application**

| **Application** | **MARS items (n)** | **Krippendorff’s α (ordinal)** |
| --- | --- | --- |
| AFib Manager | 18 | 0.27 |
| AFib 2gether | 18 | 0.59 |
| cliexa-PULSE | 18 | 0.45 |
| AFibLife | 18 | 0.36 |
| Health Storylines | 18 | 0.69 |
| Overall (pooled) | 90 | 0.53 |

MARS = Mobile Application Rating Scale. Krippendorff’s α was calculated on the two reviewers’ independent item-level ratings using the ordinal method (irr package; R version 4.3.3). Eighteen MARS quality items were rated per application (Engagement, 5; Functionality, 4; Aesthetics, 3; Information, 6); the Evidence base item was rated “not applicable” for all applications and was excluded, giving 90 pooled item ratings. Final reported MARS scores were determined by consensus. Krippendorff’s benchmarks regard α ≥ 0.80 as reliable and α between 0.667 and 0.80 as supporting only tentative conclusions; most coefficients reported here fall below 0.667, so, given the small number of items per application, between-app differences in α should be interpreted with caution.

**Supplemental Appendix 1. Mobile Application Rating Scale (MARS) scoring framework**

Reproduced/adapted with attribution from Stoyanov SR, Hides L, Kavanagh DJ, Zelenko O, Tjondronegoro D, Mani M. Mobile App Rating Scale: a new tool for assessing the quality of health mobile apps. JMIR mHealth uHealth 2015;3:e27 (open access, CC BY). Each quality item is scored 1 (Inadequate), 2 (Poor), 3 (Acceptable), 4 (Good), 5 (Excellent); “not applicable” is permitted for Information-section items where noted. Section (domain) scores are the mean of completed item scores; the app quality mean score is the mean of the four objective domain scores. Full response-option wording for each item is available in the original MARS publication (Stoyanov et al., 2015).

**Section A — Engagement**

1. Entertainment — is the app fun/entertaining to use (eg, gamification)?

2. Interest — is content presented in an interesting way?

3. Customization — does it provide/retain necessary settings and preferences?

4. Interactivity — does it allow user input, feedback, prompts/reminders?

5. Target group — is content appropriate for the target audience?

**Section B — Functionality**

6. Performance — do features and components work accurately and responsively?

7. Ease of use — how easy is it to learn and operate the app?

8. Navigation — is movement between screens logical and consistent?

9. Gestural design — are taps/swipes/scrolls consistent and intuitive?

**Section C — Aesthetics**

10. Layout — are arrangement and size of on-screen elements appropriate?

11. Graphics — quality/resolution of buttons, icons, and visuals.

12. Visual appeal — overall visual quality of the app.

**Section D — Information**

13. Accuracy of app description (in store) — does the app contain what is described?

14. Goals — does the app have specific, measurable, achievable goals? (N/A permitted)

15. Quality of information — is content correct, well written, relevant? (N/A permitted)

16. Quantity of information — is coverage comprehensive but concise? (N/A permitted)

17. Visual information — are charts/graphics/instructions clear and correct? (N/A permitted)

18. Credibility — does the app come from a legitimate/credible source?

19. Evidence base — has the app been trialed/tested in the literature? (Excluded from scoring in this study; rated “not applicable” for all applications.)

**Section E — Subjective quality (reported separately; not included in the objective mean)**

1. Would you recommend the app to people who might benefit from it?

2. How many times would you use it in the next 12 months if relevant?

3. Would you pay for the app?

4. Overall star rating.
